# Isolation and characterization of a novel S1-gene insertion porcine epidemic diarrhea virus with low pathogenicity in newborn piglets

**DOI:** 10.1080/21505594.2024.2397512

**Published:** 2024-09-16

**Authors:** Mingjun Su, Yutao Wang, Junfang Yan, Xiangwen Xu, Huihua Zheng, Jiongze Cheng, Xiaoxu Du, Yijia Liu, Jiale Ying, Yulin Zhao, Ziqi Wang, Xing Duan, Yang Yang, Changyong Cheng, Zhihui Ye, Jing Sun, Dongbo Sun, Houhui Song

**Affiliations:** a Key Laboratory of Applied Technology on Green-Eco-Healthy Animal Husbandry of Zhejiang Province, Zhejiang Provincial Engineering Research Center for Animal Health Diagnostics & Advanced Technology, Zhejiang International Science and Technology Cooperation Base for Veterinary Medicine and Health Management, China-Australia Joint Laboratory for Animal Health Big Data Analytics, College of Animal Science and Technology & College of Veterinary Medicine of Zhejiang A&F University, Hangzhou, Zhejiang Province, China; b Ningbo Creator Animal Pharmaceutical Co. Ltd, Ningbo, Zhejiang Province, PR China; c Laboratory for the Prevention and Control of Swine Infectious Diseases, College of Animal Science and Veterinary Medicine, Heilongjiang Bayi Agricultural University, Daqing, PR China

**Keywords:** Porcine epidemic diarrhea virus, S gene, mutation, insertion, pathogenicity

## Abstract

Porcine epidemic diarrhea virus (PEDV) causes diarrhea and vomiting in piglets, leading to a mortality rate of 100%. Due to the high frequency of mutation, it is important to monitor the evolution of PEDV and develop potential vaccine candidates. In this study, two PEDV strains (ZJ2022 and ZQ2022) were identified by PCR. These strains were subsequently isolated, and their genome sequences, growth characteristics, and pathogenicity were compared. Phylogenetic and recombination analyses revealed that both strains belonged to GIIa-subgroup, and ZQ2022 was identified as a recombinant strain derived from ZJ2022. Further sequence analysis showed that the ZJ2022 strain had a modified top region of the S1 protein due to a three amino acid insertion (T380_Y380insGGE) in the S1 gene. According to the virus growth curve, ZJ2022 exhibited better cellular adaptation than ZQ2022, with higher viral titers from 8 hpi to 24 hpi. Additionally, ZQ2022 exhibited a high level of pathogenicity, causing severe diarrhea in piglets at 36 hpi and a 100% mortality rate by 96 hpi. In contrast, ZJ2022 showed lower pathogenicity, inducing severe diarrhea in piglets at 60 hpi, with a mortality rate of 60% at 96 hpi and 100% at 120 hpi. In summary, our findings provided evidence of the undergoing mutations in Chinese PEDV strains. Furthermore, the S gene insertion strain ZJ2022 exhibited strong cellular adaptability and low pathogenicity, making it a potential candidate strain for vaccine development.

## Introduction

Porcine epidemic diarrhea virus (PEDV) is a member of the *Alphacoronavirus* genus and is classified as an enveloped, single-stranded, positive-sense RNA virus [1]. PEDV infection elicits severe clinical symptoms in piglets, such as acute watery diarrhea, vomiting, and dehydration, with a 100% mortality rate [2,3]. PEDV identification as the causative agent of porcine epidemic diarrhea (PED) dates back to 1978 [4]. Since the initial outbreak of PED in multiple pig farms in southern China, the disease has subsequently spread various provinces, causing significant financial losses for the Chinese pork industry [5–8]. Currently, PEDV infection is widespread in swine-farming nations across Asia, Europe, and North America [9]. The occurrence and recurrence of PEDV have led to profound financial losses and pose notable public health concerns globally.

PEDV encodes a range of proteins, including sixteen non-structural proteins (nsp1-16), four structural proteins (spike (S), envelope (E), membrane (M), and nucleocapsid (N)), and an accessory protein ORF3. These proteins are essential for the virus to counteract the host’s natural immune response and regulate the host cell microenvironment [10–13]. S protein can be divided into two subunits, S1 and S2, with S1 responsible for binding to host cell surface receptors and S2 facilitating viral-cellular and cellular-membrane fusion [14]. The continuous emergence of PEDV variant strains is attributed to the S gene mutations (including point mutations, insertions, and deletions) [15–17]. Global PEDV strains are classified into two main groups: GI group (classical strains) and GII group (variant strains). The GI group further includes subgroups GI-a and GI-b, while the GII group encompasses subgroups GII-a, GII-b, and GII-c [18]. Moreover, global PEDV strains can also be categorized based on the mutations in the PEDV S gene, including S-INDEL strains (low pathogenicity) and Non S-INDEL strains (high pathogenicity) [15,19,20]. Different subtypes of PEDV strains demonstrate varying levels of pathogenicity, a characteristic closely associated with viral mutations, especially those in the S gene [21–23]. These mutations can significantly affect the ability of virus to invade host cells and hinder the production of host-neutralizing antibodies [24–26]. Presently, the predominant strains of PEDV in China are primarily GII-a and GII-b, characterized by continuous mutations in their S genes, resulting in limited cross-protection among different strains [9,17,18,27]. Therefore, it is crucial to consistently monitor the evolution of PEDV in China and stockpile vaccine strain candidates.

In this study, two previously unidentified PEDV strains from Zhejiang province of China were isolated. The complete genome sequences of these strains were obtained, and subsequent analysis was conducted to examine their genetic evolution, genome sequences, *in vitro* biological characteristics, and *in vivo* pathogenicity. The findings from this research contribute to a deeper understanding of PEDV evolution within China, and the study serves as a valuable reference for the clinical control of PEDV infection in pig farms.

## Materials and methods

### Ethical declarations

All animal experiments were approved by the Animal Experiment Ethical Committee of Heilongjiang Bayi Agricultural University (Permit Number: DWKJXY2023094; Ethical approval date: 2023/5/27) in accordance with the Regulations for the Administration of Affairs Concerning Experimental Animals.

### Cells and antibody

The Vero E6 (ATCC number: CRL-1586) and LLC-PK1 (ATCC number: CL-101) cells were grown under a humidified atmosphere of 5% CO_2_ at 37°C in Dulbecco’s modified Eagle’s medium (DMEM) containing 10% fetal bovine serum. Mouse monoclonal antibody against the N proteins of PEDV was purchased from QIANXUN Biological (Cat. No: Ab009).

### Viral isolation and identification

PEDV-positive fecal samples were chosen for viral isolation. The identification of the PEDV-positive samples following the methods described by Wang et al. (2016) [16]. Viral isolation was conducted in accordance with the PEDV isolation protocol described by Yang et al. (2020) [28]. Briefly, confluent monolayers of Vero E6 cells were cultured in 6-well plates and subsequently rinsed twice with post-inoculation medium consisting of DMEM supplemented with 5 µg/mL Trypsin-EDTA (Gibco, Cat. No: 25200072). The cells were then inoculated with 0.2 mL of sample mixed with 1.8 mL of post-inoculation medium. After incubating for 2 hours at 37°C with 5% CO_2_, the inoculum was aspirated and replaced with 3 mL of post-inoculation medium per well. Once a 70% cytopathic effect (CPE) had been observed, the plates underwent three freeze-thaw cycles. The virus was then harvested from the supernatant for subsequent propagation or stored at −80°C. The isolated viruses were then identified using an indirect immunofluorescence assay (IFA) and electron microscopy (EM). In the IFA experiment, Vero E6 cells were exposed to PEDV isolates at a 0.1 multiplicity of infection (MOI) for 36 hours. The cells were fixed with 4% paraformaldehyde for 10 min, permeabilized with 0.1% Triton X-100 in PBS for 5 min, and then blocked with 5% skim milk for 2 hours at room temperature. Subsequently, the cells were treated with primary monoclonal antibodies (dilution, × 500) targeting the Nucleocapsid (N) protein of PEDV for 1 hour at 37°C, followed by incubation with Alexa Fluor 555 donkey anti-mouse IgG (H+L) (Invitrogen, A31570). The cells were examined under a fluorescence microscope. In accordance with the methodology described by Yang et al. (2020) [28], the samples for EM underwent negative staining. Viral particles were isolated by ultracentrifugation at 30,000 ×g for 30 minutes, followed by negative stained with a 2% phosphotungstic acid solution with a pH of 7.0. The negatively stained samples were analyzed using a Hitachi-7650 transmission electron microscope (Hitachi, Ltd., Tokyo, Japan). The PEDV isolates were nominated as strain ZJ2022 and ZQ2022.

### Genome sequencing and phylogeny analysis

The complete genomes of PEDV strains ZJ2022 and ZQ2022 were obtained using the methodology described by Yang et al. (2020) [28]. The process involved RNA extraction and cDNA synthesis following the protocols outlined by Wang et al. (2016) [16]. The complete genome sequences of PEDV strains ZJ2022 and ZQ2022 were submitted to the GenBank database (https://www.ncbi.nlm.nih.gov/genbank/).

To genotype the PEDV strains ZJ2022 and ZQ2022, 202 complete PEDV genomes (Table S1) and 365 S genes (Table S2) were obtained from the NCBI nucleotide database as reference sequences. These reference sequences covered subgroups GI-a, GI-b, GII-a, and GII-b. The genomic sequences of the PEDV reference strains were used to create a neighbor-joining phylogenetic tree with the ClustalX alignment tool in the MEGA 6.06 software package [29]. Neighbor-joining phylogenetic trees were built using the p-distance model, 1000 bootstrap replicates, and default parameters in MEGA 6.06 software. The resulting trees were pruned and re-rooted using Interactive Tree Of Life software version 4.2.3 (https://itol.embl.de/), an online tool for visualizing circular trees and annotations [30]. Furthermore, similarity plots of the genomes of the PEDV strains identified in this study were generated using the sliding window method implemented in the SimPlot, v.3.5.1 package [31].

### Recombination analysis

For the analysis of recombinant sequences, seven recombination detection algorithms (RDP, GENECONV, BootScan, MaxChi, SiScan, 3Seq, and Chimaera) were applied using the recombination detection program (RDP v.4.83) with default settings. A threshold p-value of 0.01 was utilized to detect potential recombinants, following the methodology outlined by Martin et al. (2015) [32]. Additionally, SimPlot v.3.5.1 was used to further confirm sequence similarity and potential recombination events, as previously described by Lole et al. (1999) [31].

### Sequence analysis of the S1 genes of PEDV

A multiple sequence alignment tool from DNAMAN 6.0 software (Lynnon BioSoft, Point-Claire, Quebec, Canada) was utilized to align 1267 PEDV S1 gene sequences (Table S3). The resulting sequence variation was analyzed following the nomenclature system proposed by den Dunnen et al. (2001) [33]. Divergence analysis of the S1 protein of PEDV was conducted using TBtools software (v1.127) [34].

### Molecular modeling and analysis of the S1 protein of PEDV

The dominant S1 amino acid sequence of 1269 PEDV S1 gene sequences were aligned (Table S3) and then chosen for investigation of the effect of PEDV strains of ZJ2022 and ZQ2022 on the structure of the PEDV S1 protein. The predicted tertiary structures of the S1 region were modeled using the open-source modeling server SWISS-MODEL (https://swissmodel.expasy.org/) from the Swiss Institute of Bioinformatics [35]. The tertiary structures of the S1 monomer were generated using the spike protein of PEDV (PDB ID: 7W6M.1) as a reference template. The PyMOL software (The PyMOL Molecular Graphics System, Version 1.7.4 Schrödinger, LLC.) was utilized to visualize and compare these modeled tertiary structures.

### Virus purification, size determination, and growth curve

The PEDV strains ZJ2022 and ZQ2022 were purified through plaque cloning in Vero E6 cells. Vero E6 cells were exposed to diluted ZJ2022 and ZQ2022 in post-inoculation medium at 37°C for 2 hours under a 5% CO_2_ atmosphere. After removing the medium, cells of each well were covered with 2 mL of post-inoculation medium containing 1.5% agarose. Once the overlaid medium solidified, the cells were cultured at 37°C with 5% CO_2_ to facilitate the plaques development. Subsequently, the cells were subjected to a 48-hour incubation period, plaques were picked, and each plaque was inoculated into Vero E6 cells for propagation of purified plaques. The previous purification was performed twice. To compare the relative sizes of plaques formed by ZJ2022 and ZQ2022, cells were fixed in a 4% paraformaldehyde solution for of 2 hours, and visualization of the plaques was achieved by staining with crystal violet.

To determine the plaques sizes formed by each purified virus, Vero E6 cells were infection with equal amounts of ZJ2022 and ZQ2022 strains. Subsequently, a plaque assay was conducted following the protocol. At 72 hours post infection (hpi), the plaques were stained with 5% (w/v) crystal violet, and their diameters were measured using Image J software. Mean diameter and standard deviation were calculated based on data from three separate experiments.

After purifying PEDV strain ZJ2022 and ZQ2022, a viral growth curve was constructed utilizing the median tissue culture infective dose (TCID_50_) method. In brief, Vero E6 cells were seeded into the wells of 96-well plates at a density of 10^5^ cells per well in 100 μL of medium and then incubated for 48 hours at 37°C with 5% CO_2_. Subsequently, the medium was replaced with 100 μL of 10-fold serial dilutions of the virus at a MOI of 0.01 in each well. The cytopathic effect was monitored at 12-hour intervals for 5 days post-inoculation. The viral titer was determined according to the Reed and Muench method [36].

### Animal experiments

Fifteen 2-day-old piglets (mean weight 1.2 kg) were selected and confirmed to be negative for PEDV, transmissible gastroenteritis virus (TGEV) and porcine rotavirus (PRoV) with the Colloidal gold rapid detection kit (BioNote, Hwaseong-si, Gyeonggi-do, Republic of Korea). These piglets were acquired from the Experimental Farm of Northeast Agricultural University, Acheng city, Harbin, China. Then, the piglets were randomly divided into three groups (five piglets per group) and housed in three separate rooms at a constant temperature of 30°C. The piglets were fed with fresh liquid milk every 4 h. The experimental phase began after a 2-days adaptation period. Piglets in the inoculation group were orally inoculated with 3 mL of DMEM containing 3 × 10^5^ TCID_50_ of PEDV strain ZJ2022 and ZQ2022, respectively, while the control group received 3 mL of virus-free DMEM. All piglets were evaluated twice daily for clinical signs and diarrhea as previously described [28,37]. The clinical mental state was scored based on the following criteria: 0 = normal; 1 = mild lethargy (slow to move, head down); 2 = moderate lethargy (able to stand, but tended to lie down); 3 = heavier lethargy (tended to lie down, but occasionally stood); 4 = severe lethargy (recumbent, moribund, death). Diarrhea severity was scored according to the following criteria: 0 = normal; 1 = soft (“cow pies”); 2 = very soft and tended to be liquid; 3 = liquid with some solid content; 4 = watery diarrhea with no solid content. Mortality rates of the piglets in each group was recorded twice daily. Piglets that died in the PEDV group were recorded, and immediately necropsied and sampled. Surviving animals were euthanized using intravenous injection of sodium pentobarbital through the ear at a dose of 150 mg per kg body weight. Euthanasia took place in a soundproof room to prevent distress in the remaining pigs. All animal experiments were conducted in separate compartments within the experimental animal house. After samples were collected, the animals were transferred to the animal experimental center of Harbin Guosheng Biotechnology Company for conducting harmless treatment. A completed ARRIVE guidelines checklist can be found in Figure 5 ARRIVE Checklist.

### Quantitative real time polymerase chain reaction (qRT-pcr) and virus binding assay

Total RNA was extracted from cells using the RNAsimple Total RNA Kit (Tiangen Biotec. Cat. No: DP419). Complementary DNA synthesis was performed with random primers using the FastKing gDNA Dispelling RT SuperMix Kit (Tiangen Biotec, Cat. No: 4992226). Relative qRT-PCR was conducted following protocols by Li et al. [38]. The cDNA was produced using specific primers (PEDV ORF3-F: 5’-GCA CTT ATT GGC AGG CTT TGT-3’; PEDV ORF3-R: 5’-CCA TTG AGA AAA GAA AGT GTC GTA G-3’) in relative qRT-PCR with CFX96 Deep Well™ Real-Time System (Bio-Rad). The qRT-PCR reaction volume was 25 μL, including 2× SYBR® Premix Ex Taq, forward and reverse primers, template DNA, and sterile water. The reaction conditions were denaturation at 95°C for 30 s, followed by 40 cycles of 95°C for 30 s, 60°C for 30 s, and 72°C for 30 s, with relative quantification analysis based on the cycle threshold method [39].

#### Virus binding assay

LLC-PK1 cells were infected with PEDV at an MOI of 1.0 for 2 hours at 4°C. After washing the cells to remove unbound virus, total RNA was extracted and reverse transcribed into cDNA. The relative levels of PEDV ORF3 gene mRNA expression was measured using qRT-PCR.

### Histopathological examinations

At necropsy, tissue samples (jejunum and ileum of piglets) from the inoculated (36 hpi) and control groups were collected separately. After fixation for 36 h in 10% formalin at room temperature, the tissue samples were processed and embedded in paraffin. The paraffin-embedded tissues were cut into 5-μm-thick sections using a microtome (Wuhan Servicebio Technology Co. Ltd., Wuhan, China). The sections were then deparaffinized with xylene, washed in decreasing concentrations of ethanol, and stained with hematoxylin and eosin for histopathological analysis.

### Statistical analysis

All statistical analyses were conducted using GraphPad Prism 8.0 software (GraphPad Software) and significance was assessed using Student’s *t*-test. A *P*-value of less than 0.05 was considered statistically significant.

## Results

### PEDV was prevalent among piglets with diarrhea in Zhejiang Province, China

**Figure 1.**
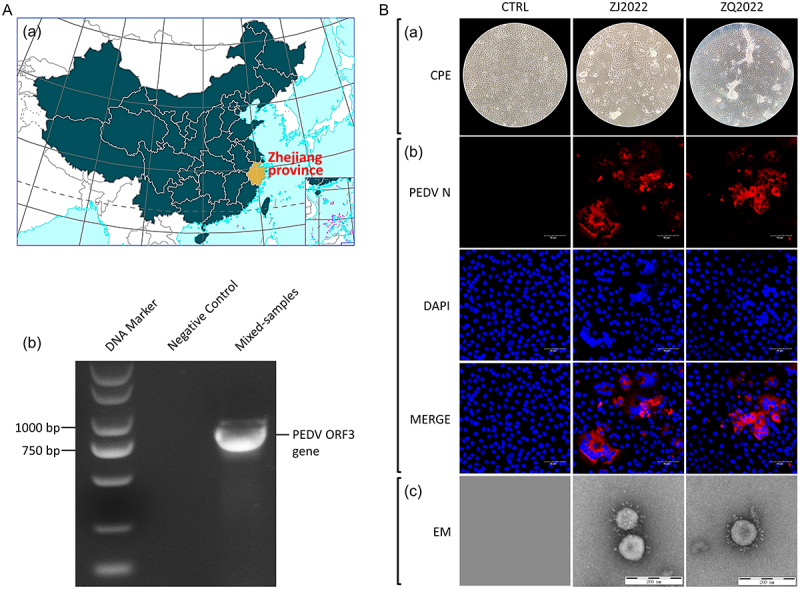
Viral isolation and identification. (A) The sample collection site was labeled with a yellow pattern (a). Identification of PEDV by PCR (b). The base map was sourced from the ministry of natural resources of china (http://bzdt.ch.mnr.gov.cn/). (b) Identification of the isolated PEDV strains ZJ2022 and ZQ2022 (P8). Cytopathic effects of the PEDV ZJ2022 and ZQ2022 strains on Vero E6 cells at 36 h post-inoculation (hpi) (100× magnification). Mock-inoculated Vero E6 cells culture showing normal cells. Virus-inoculated Vero E6 cells showing rounded and clustered cells (a). Detection of PEDV strain in Vero E6 cells by immunofluorescence (IF) staining at 36 hpi (200× magnification). If staining of mock-inoculated Vero E6 cells showing no if-positive cells. if-stained cells were visible in ZJ2022 and ZQ2022-inoculated Vero E6 cells (b). Electron microscopy of the supernatants of the ZJ2022 and ZQ2022-inoculated Vero E6 cells by using negative staining with phosphotungstic acid. Typical coronavirus-like particles were observed (scale bar = 200 nm) (c).

To investigate the prevalence of PEDV in diarrheic piglets in Zhejiang Province, a total of 34 fecal samples were obtained from symptomatic piglets in the region (Figure 1a-a). Subsequently, RNA extraction was performed on these samples followed by PCR analysis, confirming the endemic presence of PEDV in swine populations within Zhejiang Province, China (Figure 1a-b). Then, the PEDV-positive samples were selected and the virus isolation was carried out. Among the samples that tested positive for PEDV, named ZJ2022 and ZQ2022 from Zhejiang province, exhibited notable cytopathic effect (CPE) in Vero E6 cells during the initial passage 8 (P8) in comparison to the control cells (Figure 1b-a). The presence of PEDV strains ZJ2022 and ZQ2022 was further confirmed through IFA utilizing a monoclonal antibody against PEDV N protein (Figure 1b-b). EM analysis confirmed the presence of characteristic coronavirus-like particles in the suspensions of Vero E6 cells infected with strains ZJ2022 and ZQ2022, exhibiting diameters of approximately 150 nm (Figure 1b-c). The above observation substantiates that PEDV was circulating in diarrhea piglets in Zhejiang Province, and two PEDV strains were successfully isolated.

### PEDV ZJ2022 and ZQ2022 belong to GIIa-subgroup

To analyze the genome characteristics of PEDV strains isolated in this study, the genomic sequences of PEDV strains ZJ2022 and ZQ2022 were sequenced and subsequently submitted to the GenBank database with the accession numbers OQ915150 and OR061129. The genome of strain ZJ2022 and strain ZQ2022 consisted of 28,047 and 28,035 nucleotides (nt), respectively. The specific gene lengths for strain ZJ2022 were as follows: S gene (4170 nt), E gene (231 nt), N gene (681 nt), M gene (1326 nt), and ORF3 gene (675 nt). For strain ZQ2022, the corresponding gene lengths were: S gene (4158 nt), E gene (231 nt), ORF3 gene (681 nt), M gene (1326 nt), and N gene (675 nt).

**Figure 2.**
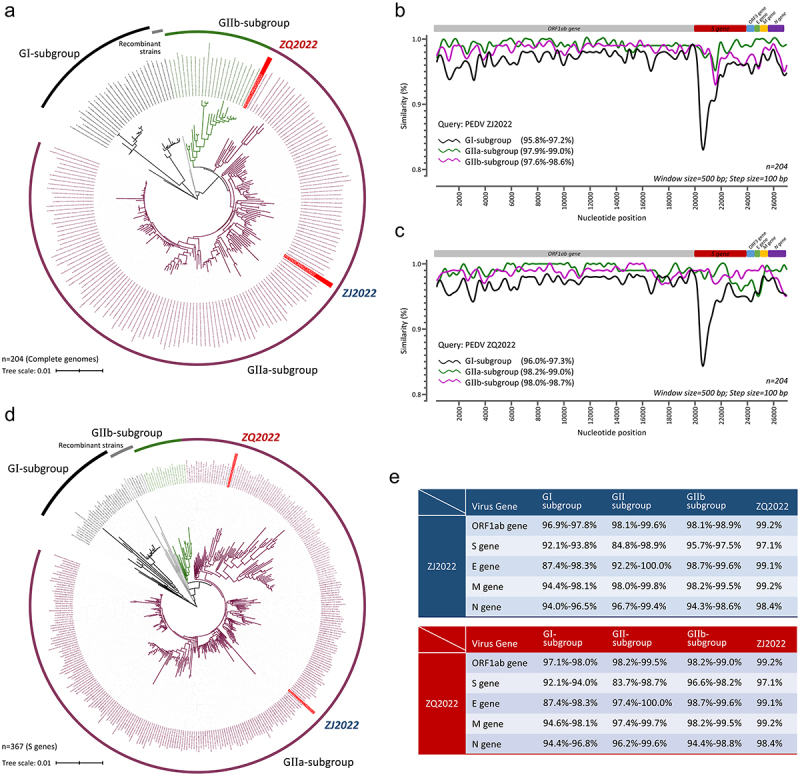
Phylogenetic analysis of PEDV strains ZJ2022 and ZQ2022. (a) Phylogenetic analysis based on the complete genome nucleotide sequence of PEDV ZJ2022 and ZQQ2022 strains and 202 reference PEDV strains. (b and c) similarity plot of the complete genome nucleotide sequence of PEDV ZJ2022 and ZQ2022 strains and 202 reference PEDV strains. The vertical and horizontal axes represent the nucleotide similarity percent and nucleotide position (bp) in the graph, respectively. (d) Phylogenetic analysis based on the S gene nucleotide sequence of PEDV ZJ2022 and ZQ2022 strains and 367 reference PEDV strains. PEDV ZJ2022 and ZQ2022 strains are marked with a red triangle. (e) Nucleotide homology of strains ZJ2022 and ZQ2022 with GI-, gIIa- and GIIb-subgroup PEDV strains, respectively.

Then, the phylogenetic tree constructed using the 204 complete genome sequences revealed that PEDV strains ZJ2022 and ZQ2022 were positioned in separate branches within the GIIa-subgroup (Figure 2a). The results of sequence alignment analysis of complete PEDV genomes showed that the nucleotide homology of ZJ2022 among GI-, GIIa-, and GIIb-subgroup strains were 95.8%-97.2%, 97.9%-99.0%, and 97.6%-98.6% (Figure 2b), respectively; the nucleotide homology of ZQ2022 among GI-, GIIa-, and GIIb-subgroup strains was 96.0%-97.3%, 98.2%-99.0%, and 98.0%-98.7%, respectively (Figure 2c); the nucleotide homology between ZJ2022 and ZQ2022 were 98.8%. Additionally, the phylogenetic analysis based on the S genes indicated that the ZJ2022 and ZQ2022 were clustered into different branches of GIIa-subgroup, consistent with above phylogenetic tree (Figure 2d). Sequence analysis revealed that the S gene nucleotide homology of ZJ2022 among GI-, GIIa-, and GIIb-subgroup strains were 92.1%-93.8%, 84.8%-98.9%, and 95.7%-97.5%, respectively; for ZQ2022, the homology among GI-, GIIa-, and GIIb-subgroup strains were 92.1%-94.0%, 83.7%-98.7%, and 96.6%-98.2%, respectively (Figure 2e). Furthermore, the nucleotide homology of ZQ2022 compared to ZJ2022 showed variations in S gene (97.1%), and with slight difference in homology of other structure genes (E gene = 99.1%, M gene = 99.2%, N gene = 98.4%) (Figure 2e). These data indicated that the prevalent PEDV strains in Zhejiang Province belong to the GIIa-subgroup, with noticeable differences in S gene among ZJ2022 and ZQ2022.

### PEDV ZQ2022 was identified as a recombinant strain evolved from ZJ2022

**Figure 3.**
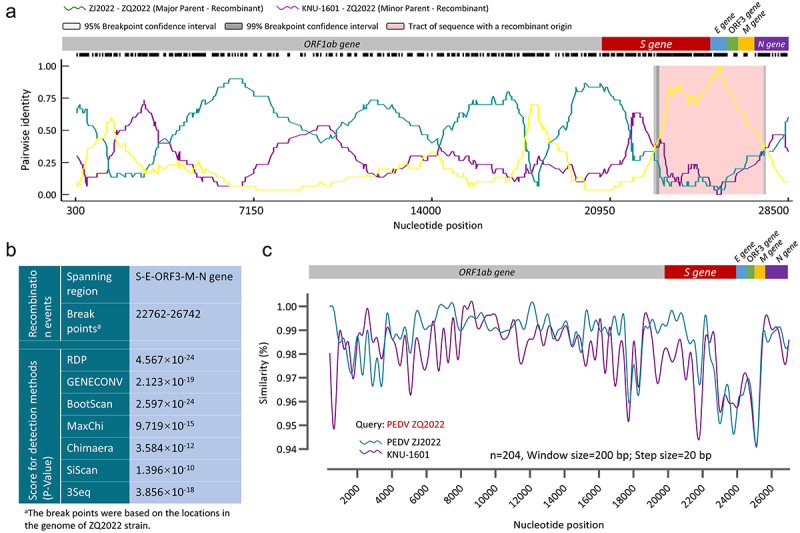
Recombination analysis of the PEDV strain ZJ2022 and ZQ2022. (a) Schematic diagram of putative recombination events detected by RDP4. The yellow line compares the major parent to the minor parent, the dark cyan line compares the major parent to the recombinant, and the purple line compares the minor parent to the recombinant. (b) Summary of recombination events in the ZJ2022 and ZQ2022 strain analyzed by RDP4. (c) SimPlot analysis was performed with ZQ2022, ZJ2022, and KNU-1601, with ZQ2022 as the query sequence. ZQ2022 (dark cyan) and KNU-1601 (purple) are identified as putative parental strains.

To determine the evolutionary relationship between the ZJ2022 and ZQ2022, recombination analysis was performed based on these two strains and the above 202 PEDV reference strains using RDP4 software. The analysis results indicate that the ZQ2022 strain possibly originated from recombination between the major parent strain ZJ2022 and the minor parent strain KNU-1601 (KY963963) from the GIIa-subgroup (Figure 3a). This conclusion was supported by seven different methods (*p*-Value of RDP = 4.567 × 10^−24^; *p*-Value of GENECONV = 2.123 × 10^−19^; *p*-Value of BootScan = 2.597 × 10^−24^; *p*-Value of MaxChi = 9.719 × 10^−15^; *p*-Value of Chimaera = 3.584 × 10^−12^; *p*-Value of SiScan = 1.396 × 10^−10^; *p*-Value of 3Seq = 3.856 × 10^−18^) (Figure 3b). To further confirm the combination events, genomic sequences of the ZQ2022 strain, ZJ2022 strain, and KNU-1601 strain were compared using SimPlotv.3.5.1. The analysis revealed that the recombination signal and breakpoints of ZQ2022 were located at positions 22,762–26742 nt, with the recombination region covering the S-ORF3-E -M-N genes (Figure 3a and 3). These findings suggest the presence of recombinant variants in the currently prevalent PEDV in Zhejiang Province, indicating that PEDV ZQ2022 may have evolved through recombination of ZJ2022.

### PEDV ZJ2022 presents a novel insertion mutation in the S1 gene

**Figure 4.**
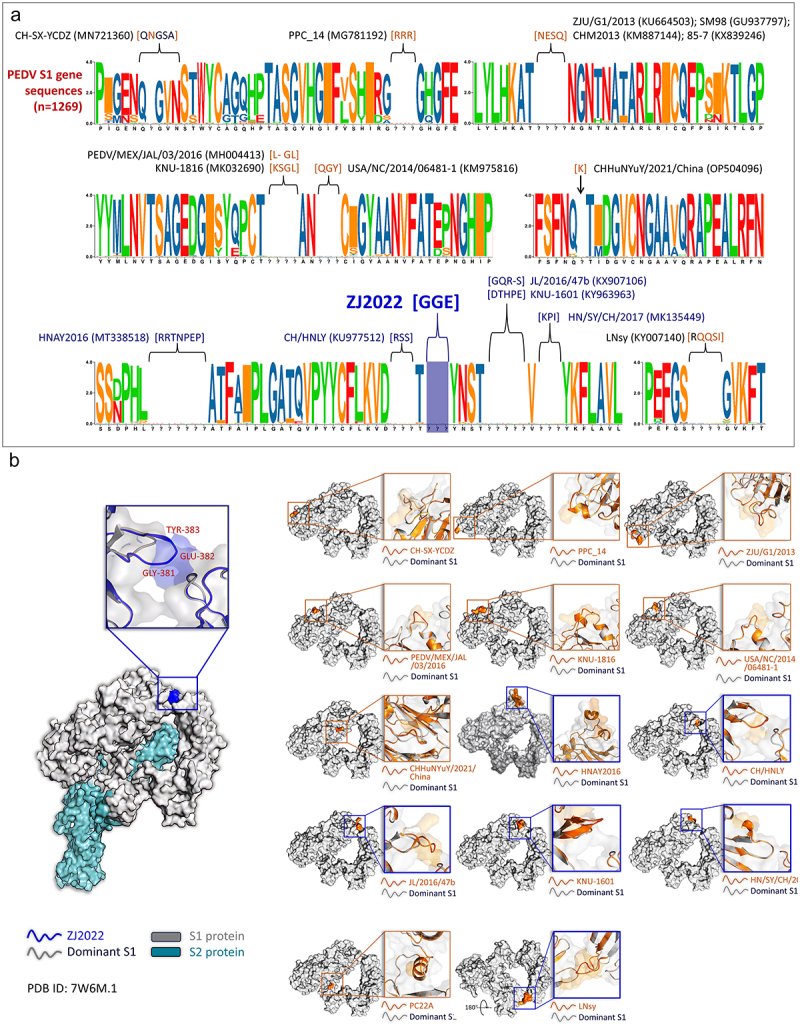
Sequence analysis of S1 proteins of the PEDV ZJ2022 strain. (a) Divergence analysis of S1 proteins of PEDV strains. (b) Comparative analysis of the predicted S1 protein modelling between S1 sequence of PEDV ZJ2022 strain (or other 13 S1 sequences of insertion PEDV strains) and dominant PEDV S1 sequence.

The S1 protein domain of coronaviruses is located in the outermost region of the virus and is highly susceptible to mutation, which is closely associated with viral infection [40]. In the case of ZJ2022, the length of the S1 gene was observed to be 9-nt longer than that of ZQ2022, indicating a deletion or insertion mutation within the S1 gene region. Therefore, the amino acid sequence alignment of the S1 protein based on these two strains and 1270 other PEDV S1 proteins from NCBI was conducted. The alignment mapping of amino acid sequences revealed that PEDV ZJ2022 had a unique insertion of 3 amino acids at 380–382 (T380_Y380insGGE), while the ZQ2022 strain showed no deletion or insertion mutations compared to all other S1 proteins. Further results indicated that insertional mutations in the PEDV S1 gene primarily occurred at positions 379–384 of the S1 dominant sequence, including that the PEDV ZJ2022 strain identified in this study, along with PEDV strains CH/HNLY (KU977512), JL/2016/47b (KX907106), KNU-1601 (KY963963), HN/SY/CH/2017 (MK135449) (Figure 4a). To investigate if the insertion of ZJ2022 S1 altered the surface structure of the S1 protein, we generated the 3D models of the S1 protein and 13 other identified insertional PEDV strains. The findings revealed that the PEDV ZJ2022 S1 gene insertion mutation (T380_Y380insGGE) changed the top region of the S1 protein surface structure, similar to PEDV strains CH/HNLY, JL/2016/47b, KNU-1601, HN/SY/CH/2017, and LNsy (Figure 4b). These results suggest that PEDV ZJ2022 represents a novel S gene insertion mutant strain, and that the insertion mutations at positions 379–384 of the S1 gene, located in the top region of the S protein, may indicate a new mutation pattern of PEDV.

### *PEDV ZJ2022 exhibits favorable* in vitro *cellular adaptability*

**Figure 5.**
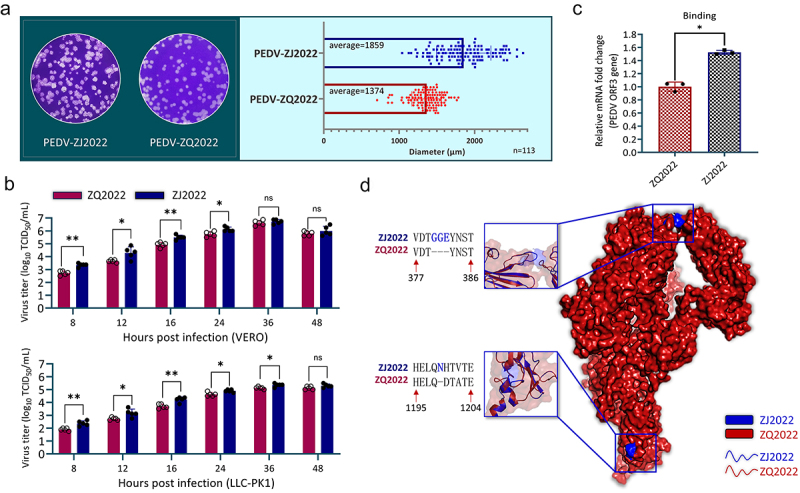
Biological characteristics of PEDV strains ZJ2022 and ZQ2022. (a) Vero E6 cells were infected with the ZJ2022 and ZQ2022 strains, respectively. The plaque size of each strain was assessed by performing the plaque assay. Left panels: representative plaque result for each strain; right panels: bar graphs displaying the averages. (b) Multi-step growth curves of PEDV strains ZJ2022 and ZQ2022 in Vero E6 and LLC-PK1 cells. (c) Viral attachment of PEDV strain ZJ2022 and ZQ2022 in LLC-PK1 cells. (d) Comparative analysis of the predicted S1 protein modelling between PEDV ZJ2022 strain and PEDV ZQ2022 strains. Statistically significant differences are indicated by * (*p* < 0.05), and ** (*p* < 0.01).

To assess the biological characteristics of PEDV strains ZJ2022 and ZQ2022, plaque sizes and growth curves were examined. The results showed that the mean plaque diameter of ZJ2022 (P8) (1859 μm) was significantly larger than that of the ZJ2022 (P8) strain (1374 μm) (Figure 5a). In addition, the multi-step growth curve revealed that ZJ2022 showed superior cellular adaptation compared to ZQ2022. The peaked viral titers of the ZJ2022 and ZQ2022 strains at 36 hpi were 6.725 ± 0.163 log_10_ TCID_50_/mL and 6.625 ± 0.197 log_10_ TCID_50_/mL, respectively. In addition, the viral titer of ZJ2022 was higher than ZQ2022 from 8 to 24 hpi, especially at 8 hpi, where the viral titer of ZJ2022 (3.35 ± 0.137 log_10_ TCID_50_/mL) was notably higher than that of ZQ2022 (2.725 ± 0.163 log_10_ TCID_50_/mL) (*p* < 0.01) (Figure 5b). In addition, the above observations were replicated in porcine-derived cells LLC-PK1 (Figure 5b). To assess differences in binding, PEDV mRNA levels were measured using qRT-PCR for the two strains. The results showed that, at the stage of viral binding, the level of virus mRNA of ZJ2022 was higher than ZQ2022 (*p* < 0.05) (Figure 5c). The S1 protein of coronaviruses is responsible for binding to the receptor, which is essential during viral entry stage. Comparison of the 3D models of the S1 proteins of ZJ2022 and ZQ2022 revealed that, in addition to the three amino acid (GGE) insertion at 380–382, ZJ2022 also has the presence of a single amino acid (N) insertion at 1199, when compared with ZQ2022 (Figure 5d). These data suggest that ZJ2022 exhibits better cellular adaptation, which may be related to its S1 gene insertion mutation.

### PEDV ZJ2022 presents low pathogenic in newborn piglets

**Figure 6.**
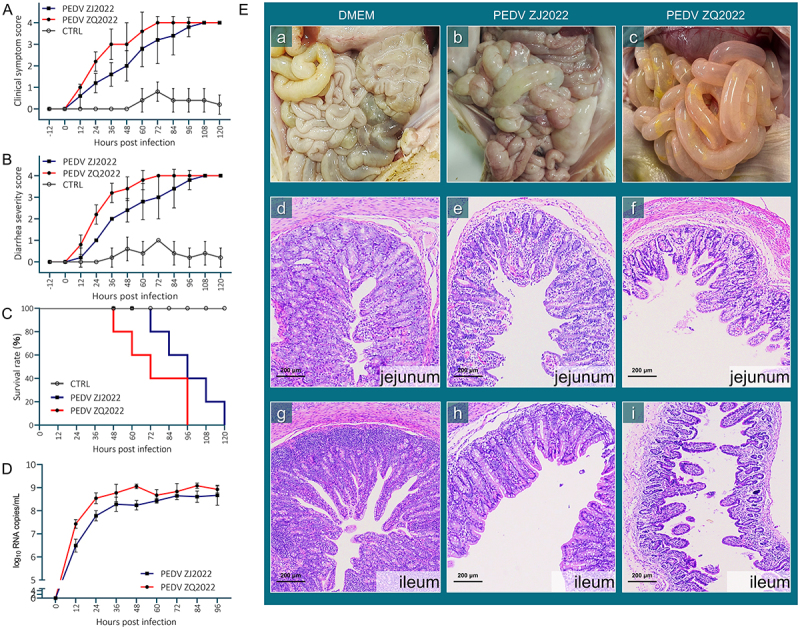
Pathogenicity analysis of PEDV strains ZJ2022 and ZQ2022. (a) Clinical symptom scores of piglets in each group. (b) Diarrhea severity score of piglets in each group. (c) Survival rate of piglets in each group. (d) Fecal viral shedding analysis of PEDV strains ZJ2022 and ZQ2022. (e) Necropsy examinations, histopathology and immunohistochemistry of the intestines of the PEDV challenge piglets at 48 hpi. Macroscopic images of intestines from ZJ2022-challenged, ZQ2022-challenged and DMEM control piglets at 48 hpi (a-c). he-stained jejunum and ileum tissue section of PEDV ZJ2022, PEDV ZQ2022-challenged and DMEM control piglets at 48 hpi (d-i).

The animal experiments showed that the severity of clinical symptoms and diarrhea was mild in the piglets of the ZJ2022-infected group when compared with piglets in ZQ2022-infected group. In ZQ2022-infected group, one (1/5) piglet appeared to have severe diarrhea at 36 hpi, and all five (5/5) piglets showed severe diarrhea and vomiting at 72 hpi and then died within 48 hpi to 96 hpi; while in ZJ2022-infected group, severe diarrhea was observed in two (2/5) piglets at 48 hpi, with the first death occurring at 72 hpi, followed by one piglet every 12 hours (Figure 6a-c). As for the mock groups, no (0/5) piglets died. The necropsy indicated the presence of extensive lesions in the intestinal tract. Viral shedding in piglets was assessed using rectal swab samples and RT-qPCR. Results indicated that those in the experimental group showed high levels of virus shed in feces (10^7.43^ to 10^9.08^ RNA copies/mL for ZJ2022; 10^6.48^ to 10^8.66^ RNA copies/mL for ZJ2022), from 12 to 96 hpi, with the ZQ2022 group having higher levels than the ZJ2022 group (Figure 6d). All piglets in the control group tested negative for PEDV. The intestines of both groups of piglets infected with PEDV exhibited distension and transparency, with ZQ2022 causing more pronounced intestinal damage in piglets compared to ZJ2022 (Figure 6e-b and Figure 6d-c). In the mock groups, no lesions or only slight lesions were observed in the piglets (Figure 6e-a). Similarly, histopathological analysis revealed that both ZJ2022 and ZQ2022 caused severe damage to the jejunum and ileum, with the ZJ2022-infected group showing slightly weaker pathological damage to intestinal tissue compared to the ZQ2022-infected group (Figure 6e-e to Figure 6e-i). In the mock groups, no damage or slight damage was observed in intestinal tissue (Figure 6e-d). These findings suggested that both ZJ2022 and ZQ2022 strains can lead to severe diarrhea symptoms in piglets, with the ZJ2022 strain exhibiting relatively milder pathogenicity to piglets.

## Discussion

Coronaviruses are single-stranded positive-stranded RNA viruses that lack the enzyme system necessary for replication, relying on host cells to complete replication, and lacking a proofreading mechanism during replication. As a result, coronaviruses are prone to mutation and recombination due to the influence of host and external factors [41]. The continuous mutation of coronaviruses frequently leads to the presence of multiple genotypes of prevalent strains, complicating prevention and control efforts [42,43]. For instance, more than 4,000 variant strains of severe acute respiratory syndrome coronavirus-2 have emerged, with the emerging variants showing increased infectivity and transmissibility [44]. PEDV is a porcine enteric coronavirus that has been globally endemic since its initial identification in the 1970’s. The first outbreak of PEDV was in southern China in 2011. Since then, the PEDV has become endemic in Chinese swine herds and continues to mutate, particularly through recombination and mutations in the S gene. These changes have affected its infectivity and pathogenicity [7,44,45]. Therefore, it is important to isolation of PEDV variant strains, analyze the pathogenicity of current epidemic strains, and stockpile vaccine candidate strains for disease prevention and control. In this study, we successfully isolated two novel GII-a PEDV mutant strains, ZJ2022 and ZQ2022, from porcine diarrhea samples in Zhejiang Province, China. Among them, ZJ2022, an S gene insertion mutant, demonstrated strong *in vitro* cellular adaptability and low pathogenicity to newborn piglets.

The *in vitro* cellular adaptability of PEDV varies significantly among different isolates [8,46,47]. Viral titer, indicating in vitro viral proliferation, is an important indicator for identifying potential vaccine strains of PEDV. In their respective studies, Yang et al. (2020) [28], Chen et al. (2019) [8], Yang et al. (2018) [47], Park et al. (2018) [48], Fan et al. (2017) [49], and Shi et al. (2017) [50], have reported the following titers for different strains of PEDV: 1.33 × 10^7^ TCID_50_/mL for the HM2017 strain at passage 15 [28], 5.44 × 10^5^ TCID_50_/mL for the FJzz1 strain at passage 5 [8], 10^6.5^ TCID_50_/mL for the QIAP1401 strain at passage 10 [47], 10^6^ TCID_50_/mL for the PED-CUP-B2014 strain at passage 40 [48], and 10^4.5^ TCID_50_/mL for the AH2012/12 strain at passage 10 [49], 10^5.5^ TCID_50_/mL for the NJ strain at passage 45 [50]. In the present study, PEDV strains ZJ2022 showed good cellular adaptability *in vitro*, with an infection titer of 10^6.725±0.163^ TCID_50_/mL for the 10th generation of viruses in Vero E6 cells, similar to that of HM2017 but higher than previous reports. These data indicate that the GII-a subgroup recombinant PEDV strain ZJ2022 holds promise as a vaccine candidate strain.

Recombination is a common phenomenon in coronaviruses and has been widely suggested as a catalyst for the emergence of novel strains, thereby significantly impacting the evolution and pathogenesis of coronaviruses [51–53]. PEDV is a globally prevalent virus, and there is a strong correlation among PEDV strains from different countries. Research by He et al. (2021) revealed that China has the most diverse PEDV lineage, with frequent occurrences of PEDV strains entering China from South Korea, Japan, and the United States [9,51,54]. There is evidence indicating that the first introduction of PEDV into China may have occurred through South Korea, possibly linked to a recombination event [55]. In this study, ZJ2022 and ZQ2022 was isolated from diarrhea piglet samples from Zhejiang Province, while KNU-1601 was identified from pig samples from South Korea in 2016. Hence, we consider the ZQ2022 strain to be a newly emerged natural recombinant strain. However, these two PEDV strains isolated in this study were derived from cell culture-adapted viruses, which could have cell-adaptation-associated mutations, and the recombination events described in this study were based on predictive analyses of recombination software, indicating limitations in the accuracy of these events. The recombination of viruses leads to alterations in their transmissibility, infectivity, and pathogenicity [52,56]. Liu et al. (2022) found that substitution of a recombinant segment from a recombinant strain inserted into the classical strain CV777 significantly enhanced the infectivity and pathogenicity of latter [45]. Similarly, PEDV ZJ2022 strain exhibited relatively low pathogenicity in newborn piglets, causing severe acute diarrhea in piglets at 60 hpi and resulting in death at 72 hpi. While ZQ2022, derived from ZJ2022, exhibited stronger pathogenicity, causing severe acute diarrhea in piglets at 36 hpi and mortality at 48 hpi with an inoculation dose of 3 × 10^5^ TCID_50_, indicating that ZQ2022 is a prevalent recombinant strain with high pathogenicity. The data above suggest that ZJ2022 may have transformed into the highly pathogenic ZQ2022 through recombinant mutation.

The S protein of coronaviruses plays a crucial role in facilitating viral entry into host cells by binding to the viral receptor. Located on the surface of the viral particle, the S protein demonstrates high variability among coronaviruses [17]. Li et al. (2021) showed that a novel PEDV isolate, named HNAY, possessed 7 amino acid insertion in its S1 gene (358–364), displaying higher pathogenicity (all piglets died within 48 hpi) compared to two other non-insertion PEDV strains (HNXX and HB), suggesting that the deletion-insertion mutation in the S protein could potentially impact the pathogenicity of PEDV in piglets [57]. Furthermore, Fan et al. (2017) identified a highly pathogenic S gene-deletion (N58 and S59) strain of PEDV JSCZ1601, which caused lethargy and diarrheic feces in neonatal pigs within 48 hours post-infection, and showed high viral loads ranging from 10^7.3^ to 10^10.1^ genomic copies/mL in the intestine contents [58]. Sun et al. (2018) reported a novel strain of PEDV LY4–98 characterized by a mutation in the S gene resulting in three distinct amino acid substitutions (L7, G8, and V9). This strain induces typical clinical symptoms in piglets at 24 hpi and typical intestinal lesions at 48 hpi [59]. Guo et al. (2024) showed that the PEDV strain CH/HLJ/18 had three amino acid deletions (at positions 57, 58, and 1389) and three unique amino acid mutations (at positions 56, 71, and 1316) in the S gene. This PEDV strain induced typical clinical symptoms of PEDV in piglets at 24 hpi, resulting in the mortality of all infected piglets within 7 days [60]. However, further comparisons with reference strains are necessary to determine if the mutations in the S gene mentioned above affect the pathogenicity of PEDV. In this study, sequence analysis showed that ZJ2022 had a unique insertion of three amino acids at 380–382 of its S1 protein (T380_Y380insGGE) compared to the 1269 PEDV S1 protein from NCBI. Nevertheless, unlike the S gene insertion strain HNAY, ZJ2020 demonstrated lower pathogenicity compared to the non-insertion PEDV strain ZQ2022. Moreover, the PEDV insertion strains identified in recent years mainly focus on the 379–384 locus of the PEDV S1 protein, including CH/HNLY (KU977512) [61], JL/2016/47b (KX907106) [17], KNU-1601 (KY963963) [62], HN/SY/CH/2017 (MK135449) [63]. The 3D structure showed that the above insertion region is located at the top of the S protein, the outermost part of the virus, and is also the region where mutations occur more frequently, indicating that the insertion mutation in the S gene represents a novel pattern of mutation in the PEDV genome.

Variations in coronavirus S proteins is responsible for differences in the tissue tropism, pathogenicity, and transmission capacity among different strains. The hydrophilicity/hydrophobicity of the S protein, along with the antigenic epitopes, phosphorylation sites, and glycosylation sites on the surface of S protein, are important factors for coronavirus infection into the host [64–66]. Bioinformatics prediction analyses indicated that the 3 insertion amino acids (T380_Y380insGGE) of the ZJ2022 S1 gene are hydrophilic amino acids, which also affect the phosphorylation and glycosylation sites and antigenic epitopes on the S protein surface (Figure S1-S4). These analyses indicate that GGE insertion could influence the function of the S protein and influence PEDV infection, requiring further investigation.

In summary, the successful isolation of PEDV strains ZJ2022 and ZQ2022 in Zhejiang province of China has revealed their classification within the variant subgroup GII-a, which is presently prevalent among pig populations in China. The PEDV strain ZJ2022 was identified as a novel strain with an S gene insertion mutation, showing a high level of adaptation to Vero E6 cells, and demonstrating relatively low pathogenicity. The PEDV strain ZQ2022 has been identified as a new recombinant strain that evolved from ZJ2022 and has shown high virulence in newborn piglets. These findings suggest that Chinese PEDV strains have a tendency for genetic recombination and S gene insertion, and PEDV strains ZJ2022 and ZQ2022 are potential candidates for vaccine development.

## Supplementary Material

Figure S4.tif

Table S1 Information on the 202 PEDV reference strains used to construct the complete genome phylogenetic tree.xlsx

The ARRIVE Guidelines Checklist.pdf

Table S3 Information on the 1267 PEDV reference strains used to S1 protein divergence analysis.xlsx

Figure S3.tif

Figure S1.tif

Figure S5 Flowchart of animal study.tif

Table S2 nformation on the 365 PEDV reference strains used to construct the S gene phylogenetic tree.xlsx

Figure S2.tif

## Data Availability

The data that support the findings of this study in this manuscript are available at Figshare (https://doi.org/10.6084/m9.figshare.25632498.v4).

## Permission to use copyrighted material

The authors are responsible for obtaining permission from the unpublished data (in revision) cited in this manuscript.
