# Supplementary figures and images for: Isolation and characterization of a novel S1-gene insertion porcine epidemic diarrhea virus with low pathogenicity in newborn piglets
Source: Virulence. 2024 Sep 16;15(1):2397512. doi: 10.1080/21505594.2024.2397512 (PMC11407387; doi:10.1080/21505594.2024.2397512)

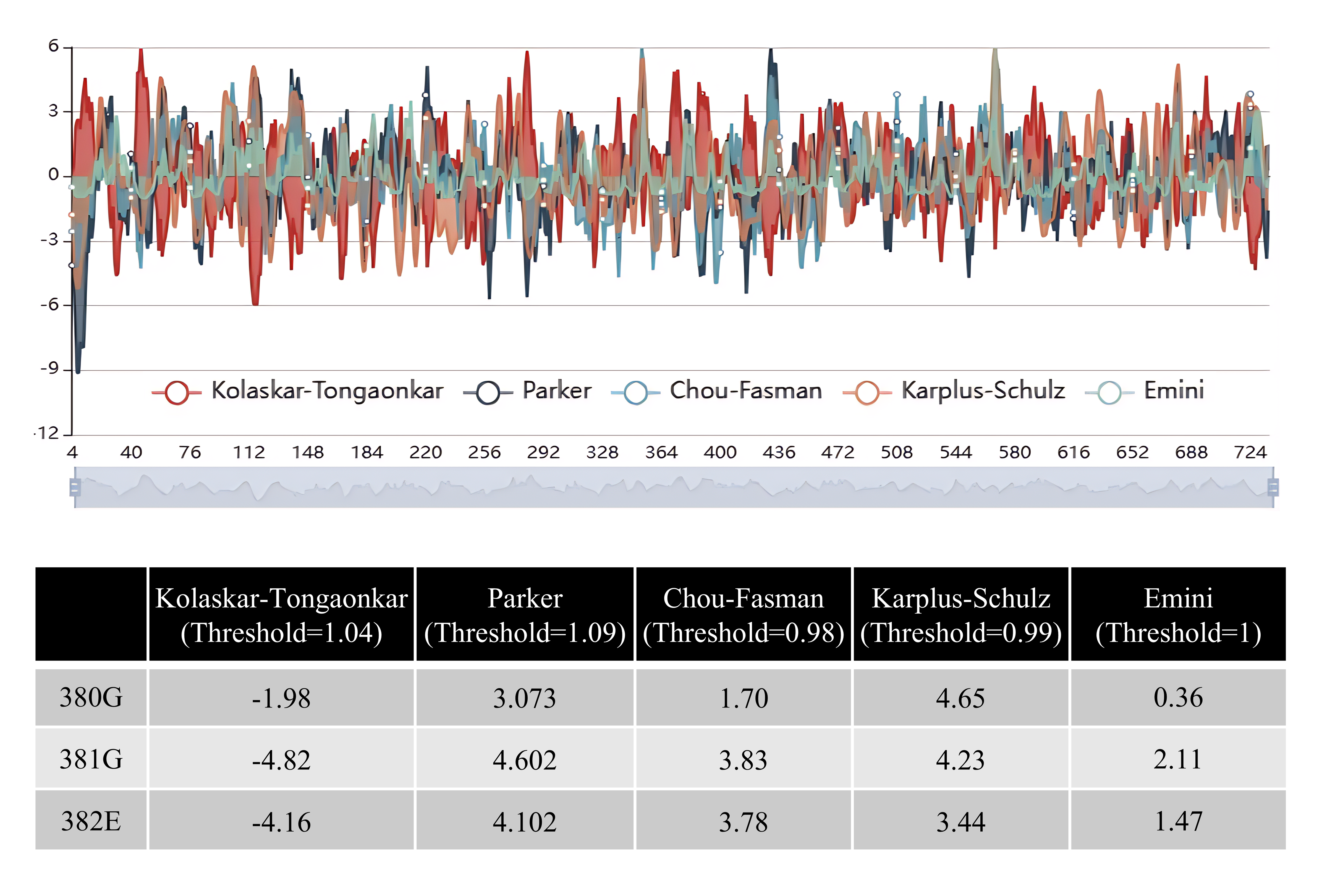

Supplement: Figure S4.tif [file KVIR_A_2397512_SM0851.tif]

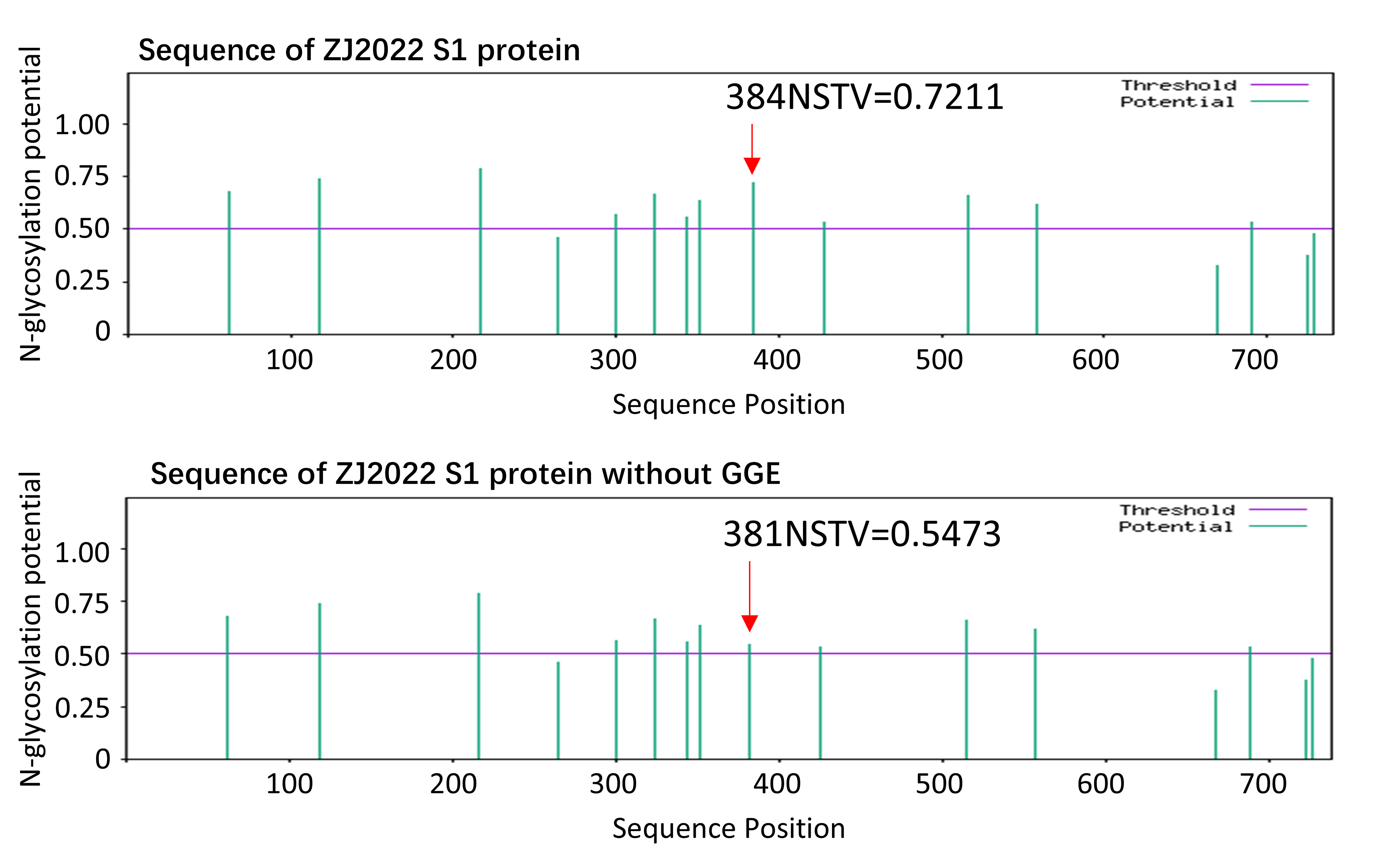

Supplement: Figure S3.tif [file KVIR_A_2397512_SM0847.tif]

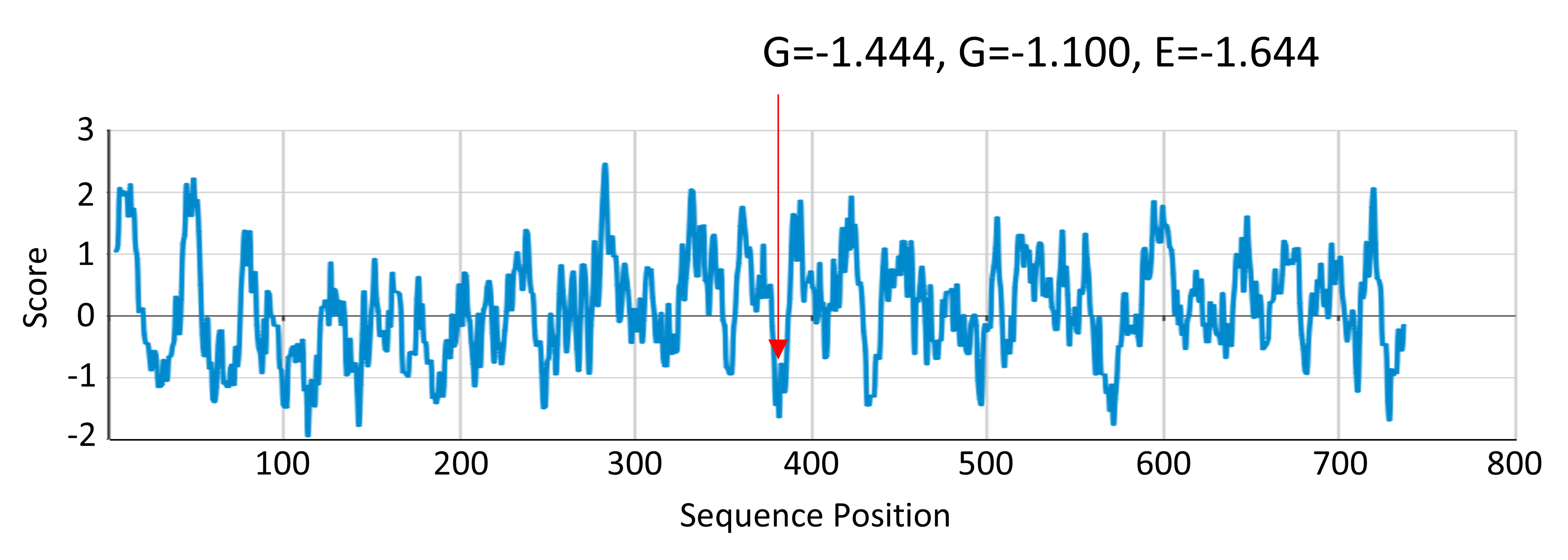

Supplement: Figure S1.tif [file KVIR_A_2397512_SM0846.tif]

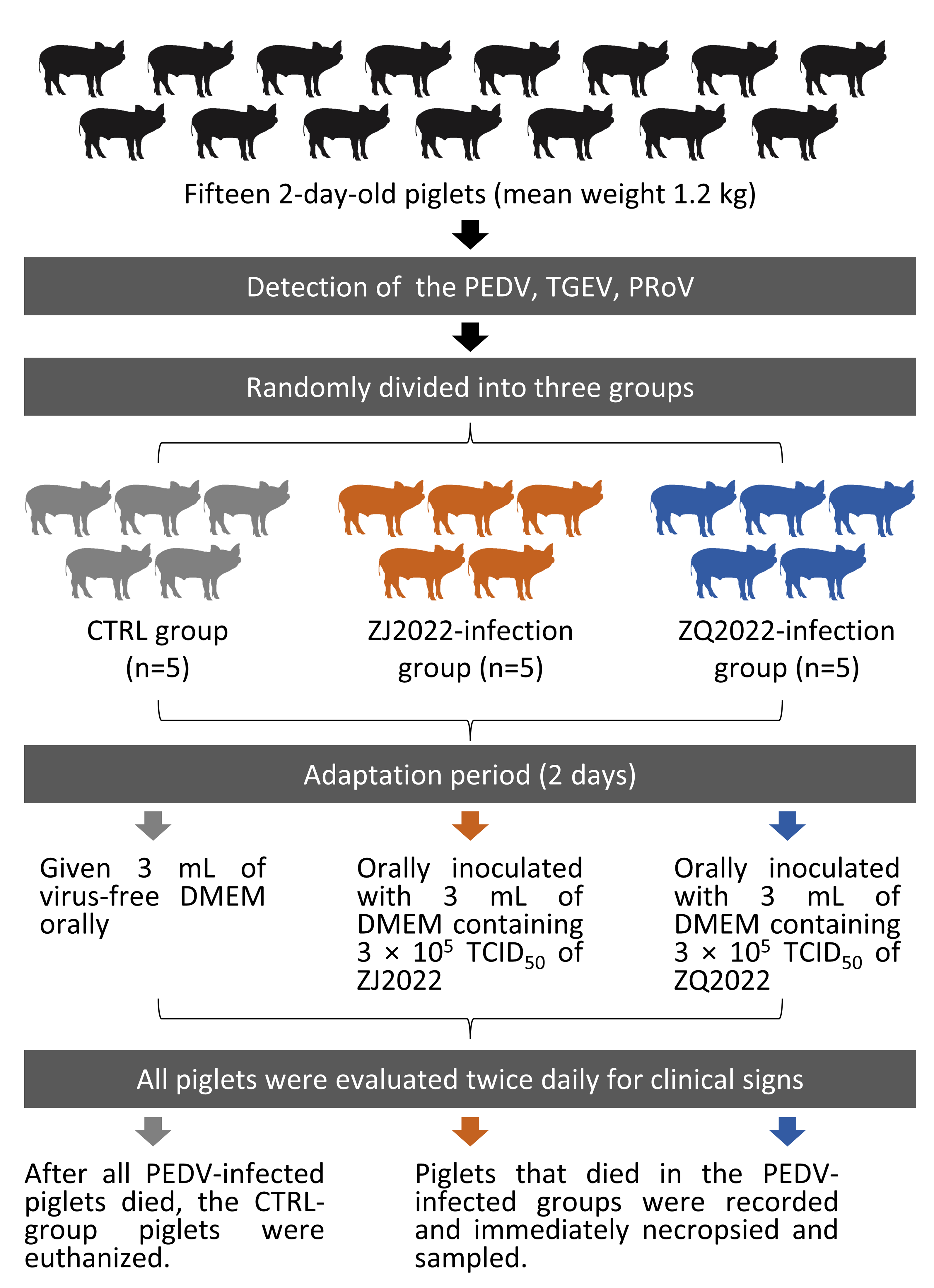

Supplement: Figure S5 Flowchart of animal study.tif [file KVIR_A_2397512_SM0845.tif]

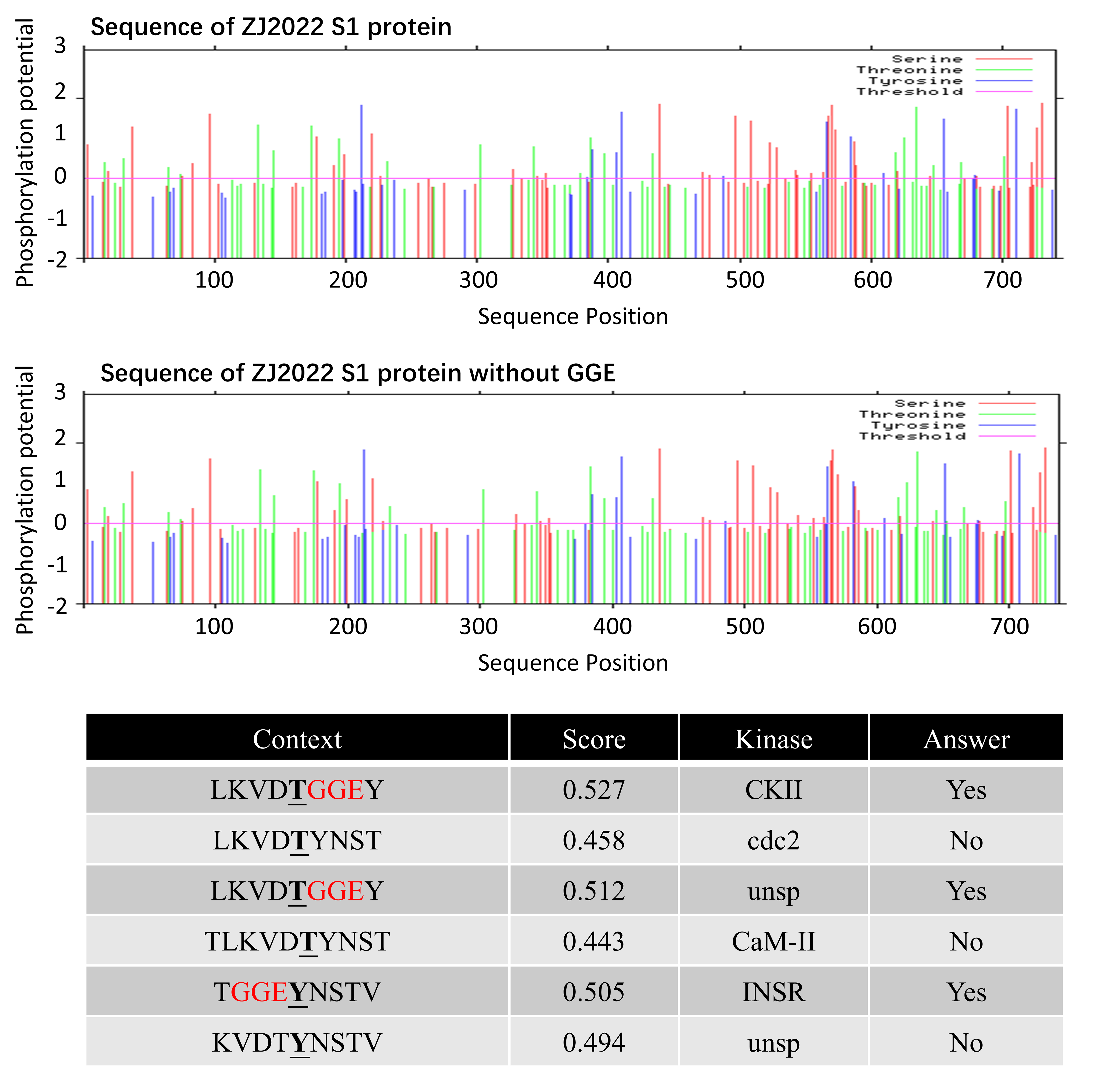

Supplement: Figure S2.tif [file KVIR_A_2397512_SM0843.tif]
